# Supplementary material for: Photonic glass for high contrast structural color
Source: Sci Rep. 2018 May 17;8:7804. doi: 10.1038/s41598-018-26119-8 (PMC5958089; doi:10.1038/s41598-018-26119-8)
Supplement: Supplementary file 1 — supplementary materials [file 41598_2018_26119_MOESM1_ESM.docx]

**Photonic glass for high contrast structural color**

*Guoliang Shang,^1,*^ Lukas Maiwald,^1^ Hagen Renner,^1^ Dirk Jalas,^1^ Maksym Dosta,^2^ Stefan Heinrich,^2^ Alexander Petrov,^1,3^ and Manfred Eich^1,4^*

*^1^Institute of Optical and Electronic Materials, Hamburg University of Technology, Eissendorfer Strasse 38, 21073 Hamburg, Germany*

*^2^Institute of Solids Process Engineering and Particle Technology, Hamburg University of Technology, Denickestrasse 15, 21073 Hamburg, Germany*

*^3^ITMO University, 49 Kronverkskii Ave., 197101, St. Petersburg, Russia*

*^4^Institute of Materials Research, Helmholtz-Zentrum Geesthacht, Max-Planck-Strasse 1, Geesthacht, D-21502, Germany*

*^*^Corresponding author: guoliang.shang@tuhh.de*

**1. First order approximation**

Here we derive the scattered-power equation using the first-order approximation in the Fraunhofer limit. The plane wave with electric field $E_{in}$ and frequency $\omega$ incident on the small volume of scattering medium $dV$, much smaller than the wavelength of light, with small permittivity contrast $\Delta\varepsilon$ generates first-order excess polarization $\varepsilon_{0}\Delta\varepsilon E_{in}$, which can be considered as a dipole emitting new spherical wave with electric field amplitude in far field [1]:

$dE=\frac{dV\Delta\varepsilon E_{in}}{4\pi}\frac{\omega^{2}}{c^{2}}\sin\gamma\frac{exp(-i\vec{k}_{s}\vec{r})}{\left| \vec{r} \right|}$ (S1)

where $\vec{k}_{s}$ is the vector with the length equal to the wavenumber of the scattered light inside the scattering medium and the direction along the scattering direction $\vec{r}$, so that $\vec{k}_{s}\vec{r}=\left| \vec{k}_{s} \right|\left| \vec{r} \right|$. The angle $\gamma$ is the angle between $\vec{k}_{s}$ and the polarization of the incident electric field $\vec{E}_{in}$. We omit the temporal dependence $\exp\left( i\omega t \right)$ in the equations. The contribution from all excited dipoles at positions $\vec{r}^{'}$ sums up to the total scattered field:

$E(\vec{r})=\frac{\omega^{2}}{c^{2}}\int\frac{\Delta\varepsilon\left( \vec{r}^{'} \right)\hat{E}_{in}\exp(-i\vec{k}_{in}\vec{r}^{'})}{4\pi}\sin\gamma'\frac{exp[-i\vec{k}_{s}^{'}\left( \vec{r}-\vec{r}^{'} \right)]}{|\vec{r}-\vec{r}^{'}|}d\vec{r}^{'3}$ (S2)

where the local polarization is now modulated with the phase $\vec{k}_{in}\vec{r}'$ of the incident wave $\hat{E}_{in}\exp(-i\vec{k}_{in}\vec{r}')$, the $\vec{k}_{s}^{'}$ vector has the length equal to the wavenumber of the scattered light inside the scattering medium and the direction along the scattering direction $\vec{r}-\vec{r}'$ and $\gamma^{'}$ is the angle between $\vec{k}_{s}^{'}$ and the polarization of the incident electric field $\vec{E}_{in}$. The origin of the vector $\vec{r}^{'}$ is defined in the vicinity of the scattering volume. In the far field several simplifications can be made, such as $|\vec{r}-\vec{r}^{'}|\approx r$, $\vec{k}_{s}^{'}\approx\vec{k}_{s}$ where $\vec{k}_{s}||\vec{r}$, and $\gamma^{'}\approx\gamma$, thus obtaining:

$E(\vec{r})=\frac{\hat{E}_{in}}{4\pi}\frac{\omega^{2}}{c^{2}}\sin\gamma\frac{exp(-i\vec{k}_{s}\vec{r})}{r}\int\Delta\varepsilon\left( \vec{r}^{'} \right)\exp[i{(\vec{k}}_{s}-\vec{k}_{in})\vec{r}')]d\vec{r}^{'3}$ (S3)

The integral now has the form of a Fourier transform. Having the total electric field and incident intensity $I_{0}=nc\varepsilon_{0}\hat{E}_{in}^{2}/2$, the intensity emitted in the far field radially into the direction $\vec{k}_{s}||\vec{r}$ can be written as

$I(\vec{r})=I_{0}\frac{\omega^{4}}{{16\pi}^{2}c^{4}}\sin^{2} \gamma\frac{1}{r^{2}}\left| \mathcal{F}\left\{ \Delta\varepsilon\left( \vec{r}' \right) \right\}\left( \vec{k}_{s}-\vec{k}_{in} \right) \right|^{2}$ (S4)

where $\mathcal{F}\left\{ \Delta\varepsilon\left( \vec{r}' \right) \right\}\left( \vec{k}_{s}-\vec{k}_{in} \right)=\int\Delta\varepsilon\left( \vec{r}^{'} \right)\exp[i{(\vec{k}}_{s}-\vec{k}_{in})\vec{r}')]d\vec{r}^{'3}$ is the three dimensional Fourier transform of the spatial function of the permittivity contrast $\Delta\varepsilon\left( \vec{r}' \right)$. Thus the scattered power can be obtained by the integral of the intensity on the spherical surface (SS) in the far field:

$P=I_{0}\frac{\omega^{4}}{{16\pi}^{2}c^{4}}\int_{SS} \sin^{2} \gamma\frac{\left| \mathcal{F}\left\{ \Delta\varepsilon\left( \vec{r} \right) \right\}\left( \vec{k}_{s}-\vec{k}_{in} \right) \right|^{2}}{r^{2}}d^{2}r_{\perp}$ (S5)

Due to the fact that $\vec{k}_{s}||\vec{r}$ and thus $\vec{r}/\left| \vec{r} \right|=\vec{k}_{s}/\left| \vec{k}_{s} \right|$, the integration over solid angle is the same in real and reciprocal space $d\Omega=(d^{2}r_{\perp})/r^{2} =(d^{2}k_{\perp})/k_{s}^{2}$. Thus the power can be also calculated by integration in reciprocal space over the surface of a sphere with radius $k_{s}$. Note that since $\vec{r}/\left| \vec{r} \right|=\vec{k}_{s}/\left| \vec{k}_{s} \right|$ the integration is over the solid angle for vectors $\vec{k}_{s}$, thus in *k*-space the integral is on the spherical surface defined by the end points of vector $\vec{k}_{s}$. We prefer to integrate in the *k*-space over vector $\vec{k}=\vec{k}_{s}-\vec{k}_{in}$. In this case the integration is done on the spherical surface of a sphere with radius $k_{s}$ and with a center shifted from the origin by vector $-\vec{k}_{in}.$This sphere can also be identified as the Ewald sphere [1]. For the unpolarized light the $\sin^{2} \gamma$ can be substituted by $g\left( \theta\right)=(1+\cos^{2} \theta)/2$, where $\theta$ is the angle between scattered $\vec{k}_{s}$ and input $\vec{k}_{in}$ wavevectors. This factor can be derived from the fact that unpolarized light has 50% of polarization always with angle $\gamma=90^{\circ}$ to the direction of scattering and another 50% with angle $\gamma=90^{\circ}-\theta$. The total scattered power is then:

$P=I_{0}\frac{\omega^{4}}{{16\pi}^{2}c^{4}}\int_{ESS} \frac{\left| \mathcal{F}\left\{ \Delta\varepsilon\left( \vec{r} \right) \right\}\left( \vec{k} \right) \right|^{2}}{k_{s}^{2}}g\left( \theta\right)d^{2}k$ (S6)

integrated over the Ewald sphere surface (ESS).

**2. Fourier transform** $\boldsymbol{C(k)}$ **of the direct correlation function** $\boldsymbol{c(r)}$

The Fourier transform $C(k)$ of the direct correlation function $c(r)$ is given by [2]:

$C\left( k \right)=\frac{24\phi}{\bar{N}}\left[ \frac{\xi_{1}+6\phi\xi_{2}+\frac{1}{2}{\phi\xi}_{1}}{\left( dk \right)^{2}}\cos\left( dk \right)-\frac{\xi_{1}+12\phi\xi_{2}+2{\phi\xi}_{1}}{\left( dk \right)^{3}}\sin\left( dk \right)-\frac{2\left( 6\phi\xi_{2}+3{\phi\xi}_{1} \right)}{\left( dk \right)^{4}}\cos\left( dk \right)+\frac{12\phi\xi_{2}}{\left( dk \right)^{4}}+\frac{12{\phi\xi}_{1}}{\left( dk \right)^{5}}\sin\left( dk \right)+\frac{12{\phi\xi}_{1}}{\left( dk \right)^{6}}(\cos\left( dk \right)-1) \right]$ (S7)

where $\bar{N}$ is the average number density of the spheres, $\phi=\left( \pi\bar{N}d^{3} \right)/6$ is the sphere packing density. The coefficients are defined as $\xi_{1}=\left( 1+2\phi\right)^{2}/\left( 1-\phi\right)^{4}$, $\xi_{2}=-\left( 1+\phi/2 \right)^{2}/{(1-\phi)}^{4}$.

**3. Examples of core-shell particle structures**

Here we consider several examples obtained by combination of four materials: air, silica, alumina and zirconia with refractive indexes of 1, 1.46, 1.68 and 2.12, respectively.

Figure S1a shows $\mathcal{F}_{m}/V$ of homogeneous zirconia sphere ($n= 2.12$) embedded in silica matrix ($n= 1.46$) with different core size. This can be implemented as zirconia spheres (diameter is $d_{c}$) with silica shells (the diameter $d$ of the shell is equal to $a$) and silica background infiltration. So, the sphere size is $0<d_{c}/d\leq1$. As can be seen from Figure S1a, $k_{m0}$ is always located at the right side of the lattice peak at $k_{lp}$ ($k_{m0} >k_{lp}$) when $d_{c}/d$ increases from 0.14 to 1.00. Similarly, the structure has the relationships of $k_{m0} >k_{lp}$ for the inverse structure of silica spheres in zirconia matrix which has a negative $\Delta\varepsilon_{1}$.

Figure S1b shows $\mathcal{F}_{m}/V$ of zirconia@silica core-shell sphere with background of air with $n_{c}=2.12$, $n_{s}=1.46$ and $n_{s}=1$. As can be seen from Figure 4b, the $k_{m0}$ is again located at the right side of the lattice peak at $k_{lp}$ ($k_{m0} >k_{lp}$) for all $d_{c}/d$. For this kind of core-shell sphere, both $\Delta\varepsilon_{1}=1.13$ and $\Delta\varepsilon_{2}=2.36$ have positive values.


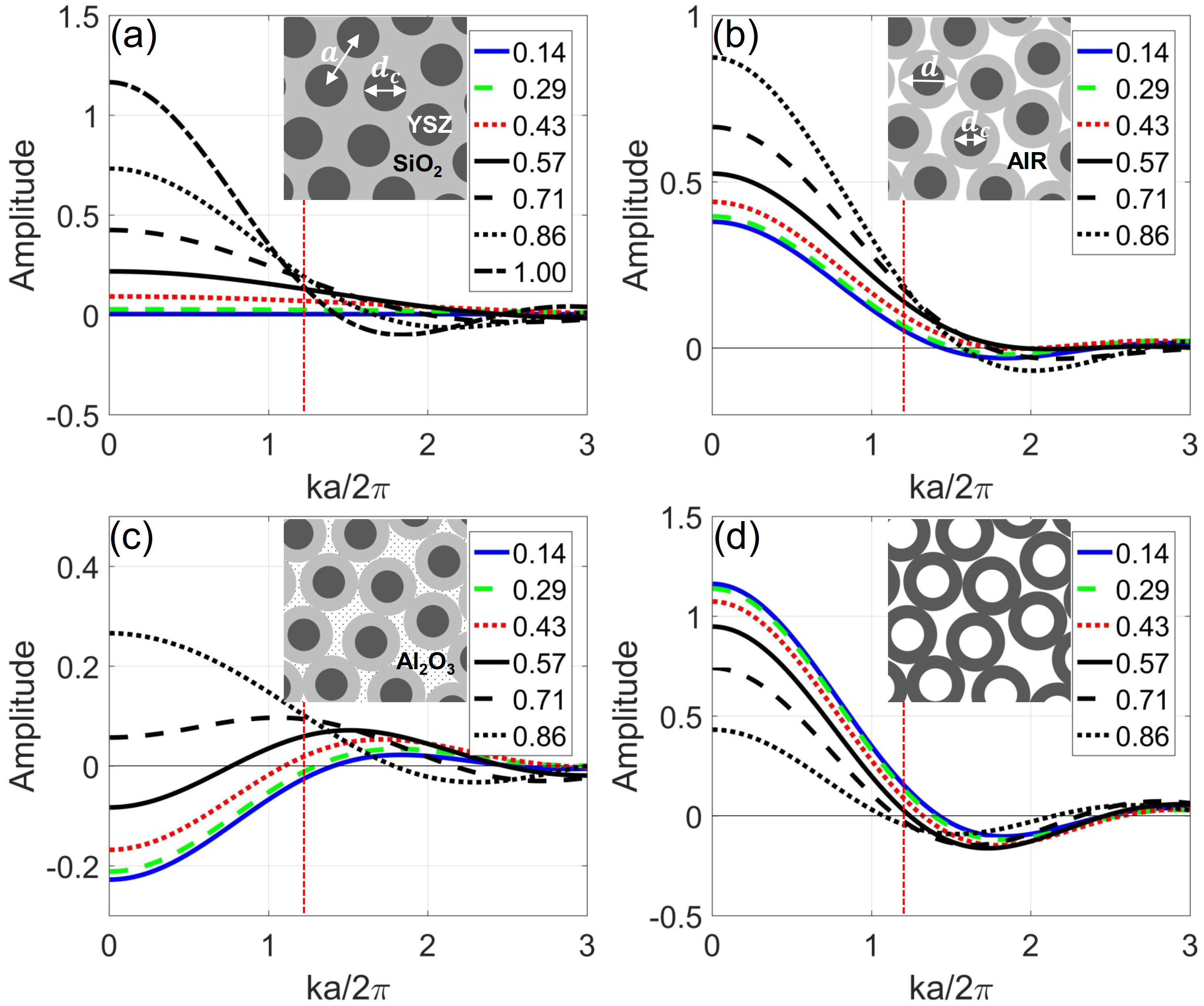


Figure S1: The Fourier transform of the motif divided by sphere volume $F_{m}/V$ with different structures. (a) Zirconia spheres embedded in the silica matrix. The structure also can be described as $n_{c}=2.12$, $n_{s}=1.46$, and $n_{b}=1.46$; (b) The zirconia@silica core-shell sphere with background of air. $n_{c}=2.12$, $n_{s}=1.46$, and $n_{b}=1$. In this case, $n_{c}>n_{s}>n_{b}$; (c) The zirconia@silica core-shell sphere with background of alumina ($n_{b}=1.68$). In this case, $n_{c}>n_{s}<n_{b}$; (d) The air@zirconia core-shell sphere with background of air. Herein, $n_{s}>n_{c}=n_{b}$. The legends in sub-images are values of $d_{c}/d$. The red vertical dash line indicates the peak position $k_{lp}=1.21(2\pi/a)$ with packing density of the spheres of 64%. The insets are the corresponding PhG illustrations.

Figure S1c shows $\mathcal{F}_{m}/V$ of the zirconia@silica core-shell sphere with background of alumina ($n_{b}=1.68$). In this case, $n_{c}>n_{s}{<n}_{b}$ with $n_{c}=2.12$, $n_{s}=1.46$ and $n_{b}=1.68$. As can be seen from Figure S1c, $k_{m0}$ can be shifted to the left side of the lattice peak if $d_{c}/d$ is in the range from 0.43 to approximately 0.65. For this kind of core-shell particles, $\Delta\varepsilon_{1}=-0.69$ and $\Delta\varepsilon_{2}=2.36$ have different signs, which can be used to shift the $k_{m0}$ to smaller $k$ positions by modifying the core size. Similarly, if $\Delta\varepsilon_{1}<0$ and $\Delta\varepsilon_{2}>0$, we can also achieve the result of $k_{m0} >k_{lp}$, for example with core-shell particles with low index core, high-index shell and background material with intermediate index.

Also, there is a special case that $\Delta\varepsilon_{1}$ and $\Delta\varepsilon_{2}$ have different signs and the core material is the same as the particle surrounding material which means $\left| \Delta\varepsilon_{1} \right|=\left| \Delta\varepsilon_{2} \right|$. Example of this structure is PhG out of hollow particles discussed in the main text. Figure S1d shows $\mathcal{F}_{m}/V$ of the air@zirconia core-shell sphere with background of air so $n_{c}=1$, $n_{s}=1.46$ and $n_{b}=1$. $k_{m0}$ can be moved to the left side when $d_{c}$ is larger than approx. 0.7.

**4. Condition for the zero point**

For the defined relationship between $\Delta\varepsilon_{1}$ and $\Delta\varepsilon_{2}$ of the core-shell particle, based on eq. 6, the zero position of the motif FT can be calculated by $\mathcal{F}_{\Delta\varepsilon_{1}}\left( k \right)+\mathcal{F}_{\Delta\varepsilon_{2}}\left( k \right)=0$, so we obtain the following equation:

$-\frac{\Delta\varepsilon_{1}}{{\Delta\varepsilon}_{2}}=\frac{\sin(k\frac{d_{c}}{2})-(k\frac{d_{c}}{2})\cos(k\frac{d_{c}}{2})}{\sin(k\frac{d}{2})-(k\frac{d}{2})\cos(k\frac{d}{2})}$ (S8)

The required situation is the zero point of motif FT located at the left side of the peak of the lattice FT, that is $k_{m0}<k_{lp}$, where $k_{lp}=1.21(2\pi/a)$ is the normalized first peak position of lattice FT as shown in Figure 2b. Because $d_{c}\leq d$, the right side of eq. S8 always has positive value when $kd/2\in(0, 1.21\pi)$, that means only if ${\Delta\varepsilon}_{1}$ and $\Delta\varepsilon_{2}$ have different sign, the requirement of $k_{m0}<k_{lp}$ can be fulfilled. Otherwise, the zero point of the motif FT is always located at the right side of the peak of the lattice FT. For the core-shell particles ($d_{c}<d$), when we know the overall particle size $d$ and the refractive index of the material, the desired $k_{m0}$ can be achieved by changing $d_{c}$ based on eq. S8.

**5. Color appearance**

Humans have three different color receptors in their eye. The vector space of discernible colors is therefore three dimensional. The projection from the infinitely dimensional space of spectral functions can be found by calculating the overlap or in other words the scalar product with the color matching functions [3]. To compare the color impression, one can set one of the three basis vector to be the brightness such that the color appearance is encoded in the remaining two variables. Such a diagram is referred to as chromaticity diagram.

Figure S2 shows the reflection spectra of (a) the solid silica and (b) the hollow zirconia particle PhGs as well as the color matching functions of the CIE 1931 XYZ color space [3]. Figure S2c shows the resulting points in the chromaticity diagram. In such a diagram a fully saturated color originating from a single wavelength can be found on the outer perimeter. A completely unsaturated color like grey or white is located in the so called white point in the center the diagram. For the solid spheres we are at at x=0.24 and y=0.25. The core-shell particles with their sharp reflection edge are further away from the white point yielding x=0.187 and y=0.092 and thus a more strongly saturated blue.


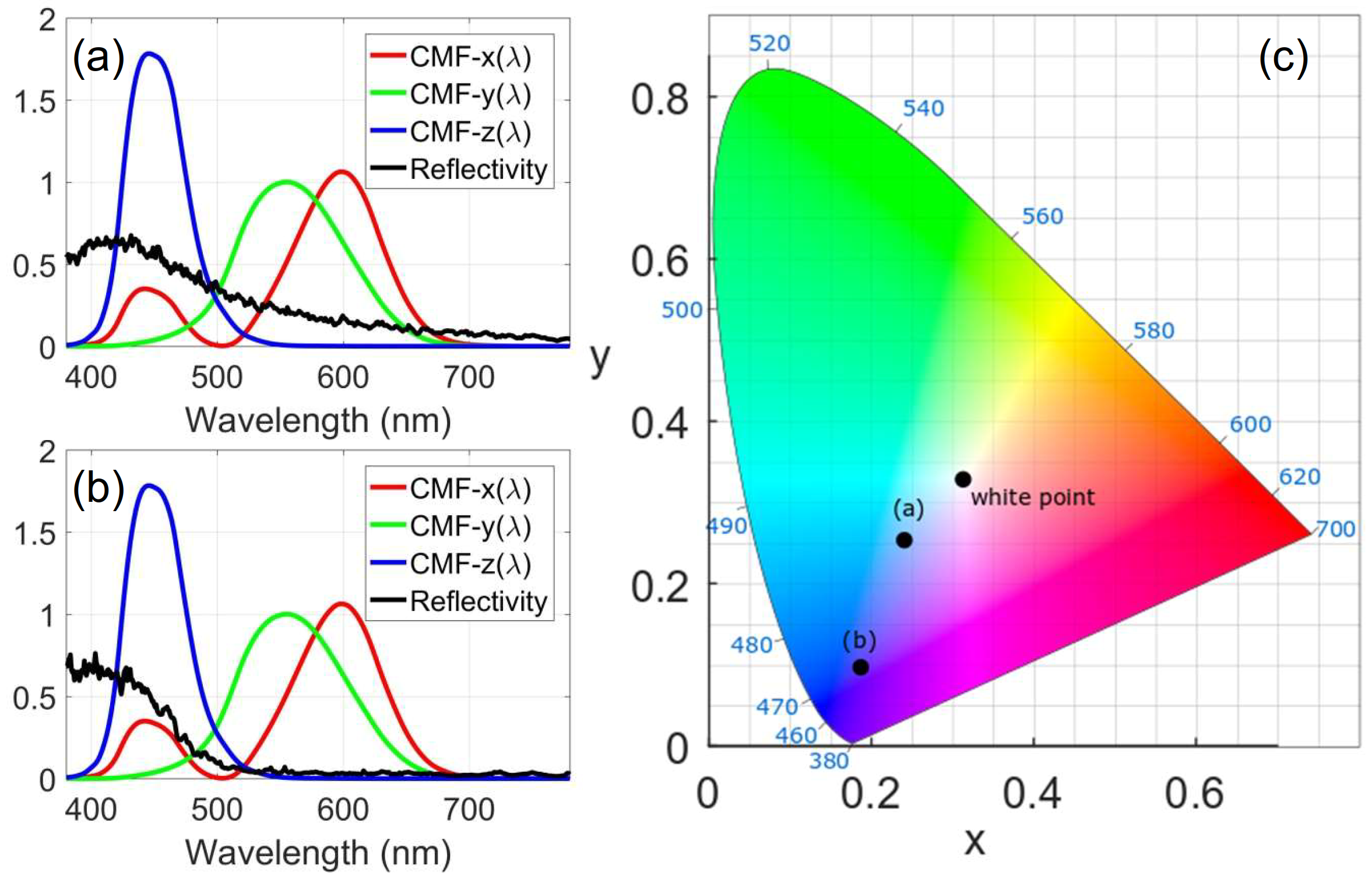


Figure S2: Reflection spectra of (a) the solid silica and (b) the hollow zirconia sphere PhGs and the matching functions (CMF) for the CIE 1931 color space. (c) Chromaticity diagram: The resulting positions of the two spectra in (a) and (b) are shown. The diagram further shows the white point and the positions of the pure colors on the perimeter indicated by the corresponding wavelengths in nm.

**References**

1. Born, M. & Wolf, E. *Principles of optics: Electromagnetic theory of propagation, interference and diffraction of light*, 7th ed. (Cambridge Univ. Press, 1999).

2. Doicu, A., Wriedt, T. & Eremin, Y. A. *Light scattering by systems of particles: Null-field method with discrete sources: Theory and programs*, (Springer, 2006).

3. Hunt, R. W. G. & Pointer, M. R. *Measuring Colour*, 2nd ed. (John Wiley & Sons, 1991).
